# Medical and Surgical Society of Baltimore

**Published:** 1878-07

**Authors:** 


### MEDICAL AND SURGICAL SOCIETY OF BALTIMORE

Dr. Wilmer Brinton exhibited a patient with the following history of syphilis from a bite:

J. W., aged 22, oyster shucker by occupation. About

Christmas, 1877, had a disagreement with one of his fellow-workmen which resulted in a fight. During the fight W. was bitten on the nose by his opponent, producing a rather severe wound, which, however, readily healed. About a month after the fight occurred the patient noticed that his nose became sore at the point where he had been bitten. This increased, becoming painful and swollen. A few weeks later the submaxillary glands became enlarged and tender, the enlargement being more decided upon the side where the bite was. There was some fever, sore throat and *malaise*, as the patient expressed it, "a bad feeling generally." About a week before applying for advice, an eruption of brownish spots, with some pimples here and there, appeared on his breast, back, arms, buttocks and thighs. When questioned regarding previous veneral troubles, he confessed to a clap about three years ago, but denied any knowledge of any ulcer of the penis. Inspection of the genitals failed to reveal any lesion or cicatrix upon the penis. The inguinal glands were very slightly enlarged. No induration along the course of the urethra, and had never suffered from stricture. Had not noticed any falling of the hair.

The site of the original injury was found to consist of an ulcer seated upon an indurated base. The submaxillary and post-cervical glands were found enlarged, and the pharynx injected. On stripping him, his trunk, arms and thighs were found covered with a well marked erythemato-papular eruption, the spots beginning to fade upon the chest and being apparently succeeded by disseminated pustules, which were most numerous around the neck. The diagnosis arrived at was that the case was one of syphilis, and the mode of infection had probably been through the bite upon the nose. It was assumed that the inflictor of the bite was syphilitic. Dr. Rohe had been called in consultation, and after examining the patient confirmed the diagnosis, but suggested "confrontation" of the patient with the man who had bitten him in order to render the evidence of the source of infection complete. On the following day the latter individual was found and examined both by Dr. Rohe and himself. An examination of the mouth revealed a large mucous patch occupying the inside of the left cheek near the labial commissure. The patch had a grayish surface, with shallow fissures running through it in various directions, and a large, firmly infiltrated base. As the evidence was deemed sufficient, no further examination was made of the man. He admitted voluntarily that some time last fall he had suffered from "the bad disorder." (Under the use of calomel locally, and bi-chloride internally, the patient, W., rapidly improved.)

Dr. I. E. Atkinson said the eruption was undoubtedly syphilitic in character, and was doubtless communicated in the manner suggested by Dr. Brinton.

Dr. Caldwell referred briefly to the manifestations of the disease in this case, and reminded the Society of the cases reported by Dr. Sims at the American Medical Association.

Dr. Rohe said he had seen this case with Dr. Brinton and had examined the man who had inflicted the troublesome bite. Although this mode of communicating syphilis was rather unusual, five cases had recently been reported by Professor Zeissl, of Vienna. One of Zeissl's cases was strikingly similar to the one just related by Dr. Brinton. The patient had been bitten by a comrade in the hand during a brawl. The wound, which was over the metacarpo-phalangeal artriculation of the left thumb, readily healed, but in a month became infiltrated and ulcerated. Secondary syphilis resulted and the man had an erythematous syphilide when he came under Prof. Zeissl's care. In another case, the initial lesion followed a kiss upon the cheek. In two cases the disease was communicated during the performance of the sexual act. The remaining case was published about nine years ago in the *Medical Times and Gazette*, and occurred in a London policeman who was bitten on the finger by a prisoneor whom he had arrested. Thorough examinations had been made in all these cases and infection in the usual way excluded.

*Osteo-Sarcoma.*—Dr. Evans related a case of supposed osteo-sarcoma of the humerus, in a boy 12 years of age.

The swelling had been noticed shortly after the boy's arm had been rudely pinched or grasped by a teacher. In the opinion of most of those present the growth of the tumor, as reported, was too rapid to be a sarcoma.

*Intestinal Hemorrhage.*—Dr. Leonard related the following cases of intestinal hemorrhage:

1. Miss M., aged 23, school teacher, alternates between two school rooms, the one kept hot, and the other quite cool; thermometer in the latter being frequently as low as 56° (Fah.) while in the former it is generally over 70°. Has had hemorrhage from the bowels during the winter of 1876–'77, with a subacute hepatitis and constipation. Gave her laxatives and ergot. The hemorrhages recurred in July, 1877, January and February, '78 (lasting six weeks), and again in March. After the last attack there was a purulent discharge from the bowels. She also complained of hemorrhoids. Would not consent to a rectal examination. The cause of these attacks was thought to be portal congestion following constant exposure to marked changes of temperature.

2. Mrs. N., aged 30, was first seen March 17, 1878. Is seven months pregnant. Has had fifteen hemorrhages from the bowel during the preceding week; is very much exhausted from loss of blood; pulse weak and compressible; 90 per minute; temperature 99.4°. Abdomen tender and tympanitic; constipation. Ordered a simple laxative.

18th. Has had no operation, but passed blood three times to-day. Ordered enema and castor oil.

19th.—9.30 A. M. Had a slight evacuation, scybalous and covered with blood. Had hemorrhage during the night and feels faint. Ordered use of bed-pan and a pill containing

| | | |
|---|---|---|
| ℞.—Hydrarg. chlor. mit, | . . . | grs. iii. |
| Aloes, | . . . . . . . | gr. i. |
| Pulu. doveri, | . . . . . . | gr. i. |
| Sig.—"At bed time." | | M. ft. pil. i. |

8 P. M. Was called to see her suddenly. She was reported to be in a convulsion, following a fainting fit. Judged the convulsion to be anæmic. Rectum healthy and empty; no piles. State of os indicates 8th month or later. Ordered fl. ext· ergt. 15 drops every 3 hours, with digitalis and stimulating diet.

21st.—9. A. M. Had several hemorrhages last night, but feels better; pill caused three free evacuations, first scybalous, last soft and normal. Ordered opium. 5 P. M.—One slight hemorrhage at 4 P. M. Feels better, but wants sleep; gave opium gr. 1 every three hours.

Midnight. Was sent for, as she again had a convulsion. Her condition was now so threatening that a consultation was determined on, with the view of inducing premature labor. Ordered chloral as a nervous sedative.

22d. Very weak; although she has had three more hemorrhages, her condition is so much improved that, with Prof. Arnold's advice, the induction of abortion was postponed. Had several labor pains during the day. Continued ergot and gave her ether for the fainting spells, of which she had several during yesterday and to-day. Has pain in the back, and several bearing-down pains, but the intervals are very long.

23d. Witnessed one of her convulsions to-day; it was not anæmic, but hysterical; while it lasted, respiration and pulse were slightly accelerated; eyes rolling and limbs rigid. The attack was preceded by a smothering sensation. Pains more frequent with discharges of blood from uterus after the pains. Stopped ergot and gave one-quarter gr. morphia, and 30 grs. potassium bromide every three hours.

24th. No hemorrhages, no convulsions, two fainting spells; labor pains increasing. Continued bromide and gave one-quarter gr. calomel, as there had been no stools, no appetite and bad taste in the mouth.

25th. Labor pains increasing in frequency and strength. Gave viburnum prunifolium, ℥ i three times a day. This was continued for three days, producing considerable disturbance of the stomach, but no appreciable good effect upon the uterine contractions. The pains continued strong but produced no dilatation. The viburnum was then stopped and the pains gradually wore away without any special treatment, and the woman finally recovered.

*Treatment of Piles by Injection of Carbolic Acid.*—Dr. Monmonier reported several cases of piles treated by the injection into the tumor of a strong solution of carbolic acid. The method, he said, was safe and effectual, and could be used in cases of persons who refused to permit the removal of the tumors by means of the knife, ecraseur or ligature. Drs. Brinton, Fiske and Leonard had tried this and found that in their hands it caused such excessive pain they would never be willing to resort to it again.

Dr. Monmonier also related a case of prolapse of the rectum, which he had successfully treated by means of the thermo-cautery.

A singular case of aural vertigo was related by Dr. Theobald. A little girl, two years of age, deaf and dumb from birth, was affected in the following manner: Frequently while running about the room playing with the other children, she fell directly backwards, striking sometimes violently on the back of the head. The attacks seemed to come without any premonition. It was believed to be due to some defect of the semi-circular canals.

Dr. Caldwell referred to a case previously shown to the Society by him, which had presented similar features.

Dr. Caldwell exhibited a patient with paralysis. Colonel ——, 57 years of age, was attacked one afternoon last November, after exertion and exposure to cold, by paralysis. The paresis extended, and on the following morning the whole of the left side was affected. He remained under care of his family physician until February last, when Dr. Caldwell was consulted. Under the use of large doses of bromide and iodide of potassium and the constant current, he rapidly improved. There is still sensory disturbance, pains about the hip and shoulder and severe headache, particularly at night. Has occasional diplopia, but no headache for five years previous to the seizure. Is very much troubled by inability to sleep. He has been married thirty-five years and is the father of one child, stated to be healthy. Syphilis was suggested as the cause of the troubles, but patient avers that he has not had sexual intercourse for 34 years.

